# Undifferentiated Embryonal Sarcoma of the Liver Presents as a Molecular Mimic of Parasitic Infection

**DOI:** 10.7759/cureus.6800

**Published:** 2020-01-28

**Authors:** Arianna Letherer, Joshua Mastenbrook, Richard A VanEnk, Laura D Bauler

**Affiliations:** 1 Medicine, Western Michigan University Homer Stryker MD School of Medicine, Kalamazoo, USA; 2 Emergency Medicine, Western Michigan University Homer Stryker MD School of Medicine, Kalamazoo, USA; 3 Biomedical Sciences, Western Michigan University Homer Stryker MD School of Medicine, Kalamazoo, USA

**Keywords:** sensitivity and specificity, predictive value of tests, enzyme-linked immunosorbent assay, sarcoma, false-positive reactions

## Abstract

Medical laboratory tests are becoming more reliable with increased specificity and sensitivity, leading to their use as definitive diagnostic tests for many medical conditions. Enzyme-linked immunosorbent assay (ELISA) tests are convenient, sensitive, and standardly used for rapid detection and quantification of antigens or patient antibodies against specific antigens. However, based upon the specificity and sensitivity of an ELISA test, the results may not be definitive for a specific disease but merely suggestive, due to potential cross-reactivity of antigens and antibodies. Here, we present a case of a 15-year-old male who presented with fever, nausea, and right upper quadrant pain. Computed tomography scan showed an 18-cm liver mass with cystic features. Biopsy results confirmed a diagnosis of undifferentiated embryonal sarcoma of the liver; however, the clinical picture was complicated by positive ELISA results for Echinococcus, Entamoeba histolytica, and histoplasmosis. Due to the absence of travel and positive ELISA result for three different infectious agents, we hypothesize that tumor molecular mimicry might have led to false-positive ELISA results in the absence of infection in this case, demonstrating a limitation of ELISA serology. Critical appraisal of all possible evidence to ensure alignment when assigning the final diagnosis is essential for optimal patient outcomes.

## Introduction

The enzyme-linked immunosorbent assay (ELISA) method is used to rapidly detect and quantify antigens and antibodies. ELISA is a convenient tool in the hospital where early detection of infection enables directed treatment. Awareness of the limitations of ELISA is a useful exercise for clinicians. We present a case in which positive ELISA serology provided misleading results. A patient with a cystic liver mass, later confirmed to be malignant, had positive serology antibody results for Echinococcus, Entamoeba histolytica, and histoplasmosis via ELISA.

## Case presentation

**Figure 1:**
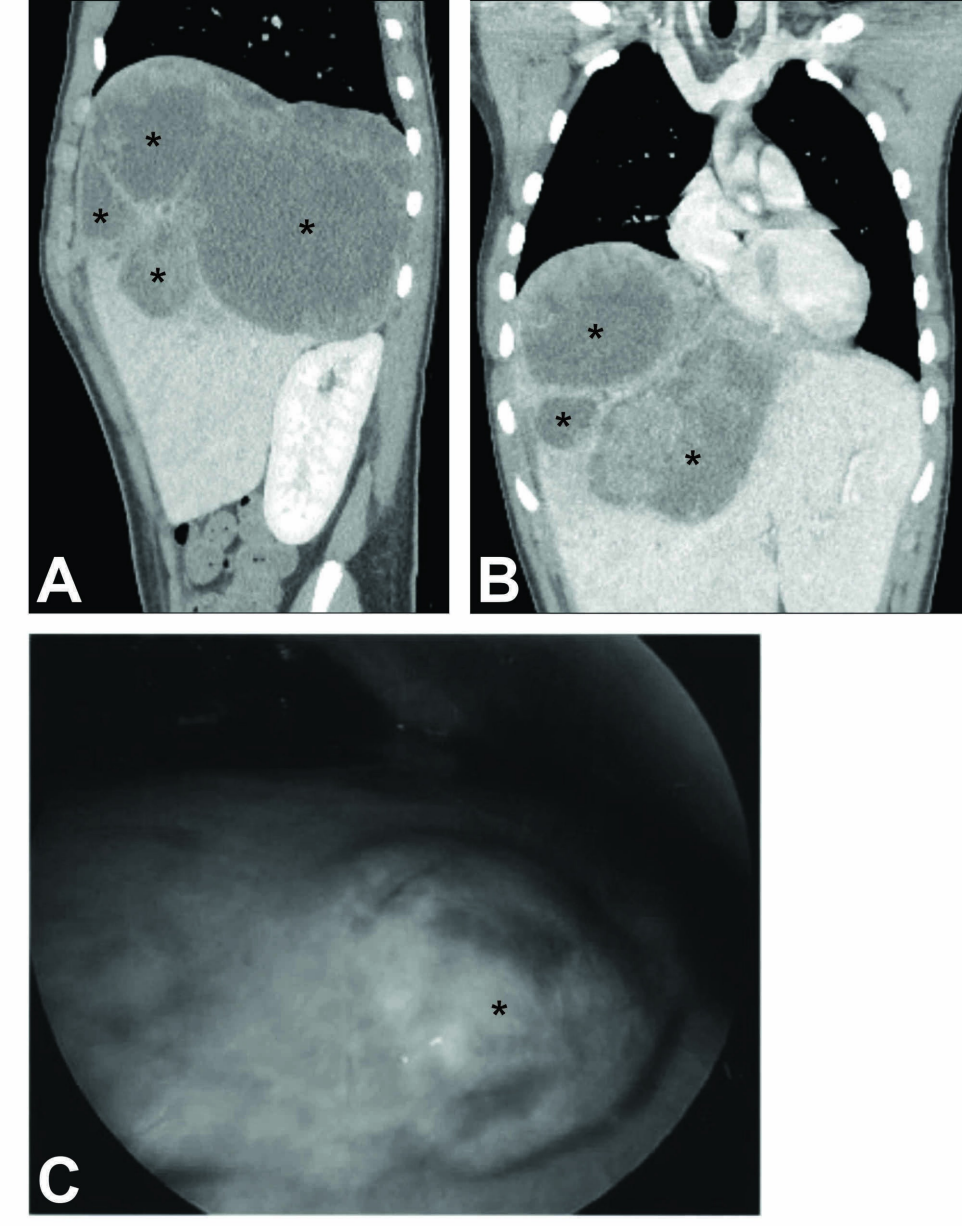
Visualization of the Hepatic Lesion (A, B) Computer tomography (CT) of the hepatic lesion. A CT scan of the abdomen and pelvis demonstrated a large (18 cm) cystic hepatic lesion shown in the sagittal (A) and coronal views (B), cysts are indicated with stars (*). (C) Intraoperative image during laparoscopic liver wedge biopsy. A portion of the hepatic mass that was biopsied can be seen (indicated by a star). The mass measured at least 18x16x14 cm and was centered within the right hepatic lobe.

A 15-year-old male, presented with fever, nausea, and three weeks of worsening right upper quadrant pain, preceded by three months of vague upper abdominal pain. Review of systems was otherwise normal. The patient had no travel history. Computed tomography (CT) imaging of the abdomen and pelvis showed an 18-cm heterogeneous hepatic mass as well as several pulmonary nodules (Figure 1A, 1B).

Given the patient’s history and the morphology of the lesion, an infectious process was initially suspected. A set of blood cultures was obtained, and serological tests including Entamoeba histolyticaantibody, serum (RIDASCREEN Entamoeba histolytica IgG, R-Biopharm AG, Darmstadt, Germany); Echinococcus antibody, IgG, serum (RIDASCREEN Echinococcus IgG, R-Biopharm AG, Darmstadt, Germany); and fungal antibodies by immunodiffusion were sent to Mayo Medical Laboratories. Initial complete blood count and blood chemistry results revealed elevated leukocytes (16,200/mm^3^), thrombocytes (536,000/mm^3^), bilirubin (2.1 mg/dL), lactate dehydrogenase (531 IU/L), gamma glyamyltransferase (119 U/L), and alkaline phosphatase (268 U/L), with normal liver transaminases and alpha-fetoprotein (AFP). Due to suspicion of a pyogenic or amoebic liver abscess, the patient was started on ceftriaxone and metronidazole.

A CT-guided needle biopsy of the liver mass taken the next day was positive for malignant cells by hematoxylin and eosin staining; however, surrounding fluid was negative for infectious agent by culture. A second set of blood cultures continued to show no growth, and antibiotics were discontinued. Subsequent laparoscopic liver biopsy (Figure 1C) led to the diagnosis of undifferentiated embryonal sarcoma, but was negative for any infectious agent. The pathology report indicated markedly pleomorphic cells with brisk mitotic activity with no differentiation and areas of hemorrhage and necrosis. Immunohistochemical staining was positive for alpha-I-antitrypsin, vimentin, and desmin; weakly positive for OSCAR focal pancytokeratin; and negative for actin and hepatocyte specific antigen; together, this was most consistent with embryonal sarcoma. The presence of lung nodules suggested stage IV metastatic disease; however, these were not biopsied.

The ELISA serological results for infectious diseases, completed after the malignancy was confirmed by biopsy, were positive for antibodies against Echinococcosis and Entamoeba histolytica, and the fungal antibody panel was positive for Histoplasma. Subsequent specific testing for Histoplasma antibody via complement fixation and immunodiffusion was negative. These confounding positive antibody results were thought to be a false positive due to a cross-reaction with the patient’s hepatic mass; as all cultures taken throughout including blood, needle biopsy, and wedge biopsy were negative.

The patient received four cycles of chemotherapy, consisting of ifosfamide and doxorubicin, followed by surgical resection of the residual tumor. Four months after his initial diagnosis, the patient underwent right hepatic trisegmentectomy along with resection of the right hemidiaphragm and mesh reconstruction. A magnetic resonance imaging scan 10 months after his initial admission revealed no evidence of disease, as did a follow-up CT scan of the chest taken 13 months after admission. 

## Discussion

ELISA is considered a sensitive and specific test and is used worldwide to evaluate a variety of infectious and immunologic processes [1]. The accuracy of an ELISA is based on two measures: (1) the sensitivity or ability to correctly identify disease positive individuals and (2) the specificity or ability to correctly identify disease negative individuals. A highly sensitive test has few false negatives, meaning fewer disease positive people will have a negative test. A more sensitive test will detect fewer molecules; however, this may decrease specificity since disease negative individuals may have a few target molecules despite being below the threshold of being positive, resulting in a false-positive result.

The sensitivity and specificity of ELISA vary by antigen. Slight variation in antigen amino acid sequence causes great variability in the sensitivity of the ELISA test. For Echinococcus, the sensitivity and specificity of an antibody detection assay have been measured at 94.2% and 82.6%, with a lower specificity due to false positives likely caused by cross-reactivity [2]. For identification of Entamoeba histolytica, ELISA accuracy was lower than microscopy and PCR [3]. Prior to clinical use, an ELISA kit is examined by the US Food and Drug Administration, who compares the clinical sensitivity and specificity of the ELISA to current detection assays. Cross-reactivity of an ELISA test cannot be fully examined. No ELISA test is 100% sensitive or specific, and for that matter, no clinical test is 100% accurate. Therefore, clinicians should be aware of the limitations of the assays used to form their clinical diagnoses. Any diagnosis should be based on the entire clinical picture, not just from a single lab test.

Undifferentiated embryonal sarcoma of the liver (UESL) is an extremely rare but highly malignant primary hepatic cancer of mesenchymal origin. It is primarily found in children aged 6-10 years, but is extremely rare with only 103 children being diagnosed between 1998 and 2012, with an overall five-year survival of 86% [4]. Early diagnosis is associated with better prognosis, making rapid diagnosis and treatment vital [4]. UESL typically presents as an abdominal mass with or without pain. Labs are often normal, although sometimes AFP, transaminase, or cancer antigen 125 will be elevated. Ultrasound displays a large solid lesion with cystic components, which frequently leads to misdiagnosis as a hydatid lesion, hepatic abscess, or a benign hepatic neoplasm [5-7]. CT is also often nonspecific and may show a hypodense mass with solid and cystic components [8]. Diagnosis can be made by pathological staining and examination of tissue, but may also be confirmed with other laboratory tests including ELISA. Subsequent ELISA testing was not completed for our patient.

In areas where Echinococcus is endemic, an infectious hydatid lesion should be higher on the differential than UESL [6]. Echinococcus antibodies persist for a prolonged amount of time beyond the active infection. Our patient had positive serology for not only Echinococcus, but also for Entamoeba histolytica and histoplasmosis with no history of exposure to any of these pathogens; therefore, we propose tumor molecular mimicry as a contributing factor to the positive ELISA results. Reports of antigenic shift in Echinococcus demonstrate that it has the potential for showing varied antigens, which complicates the identification of an antigen for use in an ELISA that is both sensitive and specific. Echinococcus cross reacts heavily with cysticercosis and there is some evidence linking false positives for Echinococcus with malignant tumors [9,10].

## Conclusions

This case serves as a cautionary tale, reminding physicians to exercise critical thinking when faced with test results that do not match the clinical picture. Although advancement of our testing capabilities has allowed for advancements in the rapid identification of infectious diseases, no test is perfect, and this case serves as a reminder of that.
